# Condensation of Gold Fillings

**Published:** 1898-07

**Authors:** A. G. Smith

**Affiliations:** Peoria, Ill., in Illinois Society.


ARTICLE II.

### CONDENSATION OF GOLD FILLINGS.

DR. A. G. SMITH, PEORIA, ILL., IN ILLINOIS SOCIETY.

For this purpose we have live blow and dead blow hand mallets, automatic mallets, neumatic mallets, mechanical mallets, electrical mallets, nearly every kind of mechanism that can be devised to deliver a blow at all, and last, but by no means least, we have hand pressure, and it is to hand pressure condensation that we must look for deliverance from many of the evils already mentioned as being connected with cohesive gold.

Several things appear in my carefully prepared experiments which are a decided surprise to me. For instance, I had always supposed that a blow from the mallet would accomplish more in the way of condensation than a thrust of a hand instrument, though I was thoroughly sure that it was not as good for the tooth. These tests, however, show conclusively that working on the same material it requires more blows than thrusts to pack a given number of pieces in a cavity of a definite size. Also I was surprised to find that hand pressure consumed less time than with automatic.

So much for the tests outside of the mouth, but it is in practical work at the chair that all operations must be finally tested, and in this connection there are many things to be said in favor of hand pressure. By this means force can be applied in any direction with almost equal facility, and this fact alone makes the number of "inaccessible" cavities very small indeed. When we become accustomed to hand pressure, there are no cavities in the mouth easier to fill than those in the distal surfaces of the superior cuspids, bicuspids, and first molars. Work on the incisor can be done from the inner or palatal side with the greatest ease both to patient and operator, as the chin does not interfere with the plugger handle, even when the mouth is partially closed.

Large buccal cavities on the molars (especially those on the third) are almost impossible to reach and fill with any instrument which depends on a blow for its efficiency, for whatever the shape of the plugger point may be, the force of a blow at the end of the instrument must always travel exactly parallel to the long axis of the handle or shaft; it is thus impossible, in many instances, to properly pack against a baccal or an anterior wall, when the cavity is situated far back in the mouth, with any mallet with which I am acquainted. Again, in hand pressure the sense of touch is not benumed and partially paralyzed by a heavy and cumbersome instrument, as with the automatic plugger, or by any rattling or clattering vibration as with many of the other forms. An important point in all goid fillings is to get them condensed evenly, and there is no way of getting information with regard to how far we have carried our condensation except through the sense of touch. We condense a filling till it feels hard enough, and we feel that it is time to add the next piece.

The lighter and more rigid the instrument with which we feel, the more accurate is our information sure to be. Who would tolerate a handle of the size, weight and looseness of an automatic plugger on an excavator or chisel? Such an instrument would be the laughing-stock of the profession should any one try to put it on the market. Who would try to prepare a cavity margin with an itstrument vibrating and chattering about as do the electrical, neumatic and mechanical mallets? Surely no one. Would we not say that such instruments as these were far too clumsy for such accurate and delicate work? Yet if there is one thing that we need to do more accurately than another it is to perfectly adapt our gold to the margins and walls of the cavity we have made.

I believe that cohesive gold is now in far more general use than the older soft variety. I have yet to see the cavity that it was possible or desirable to fill with gold at all, that, to my mind, could not be better filled with cohesive gold than with soft gold either alone or in combination with cohesive. While there are comparatively few fillings now made entirely of soft gold, many operators still cling to it as of peculiar and almost supernatural efficacy in starting fillings and in "protecting" the cervical margin. (I might remark right here that in the term "protecting the cervical margin," which is in such universal use, is a very happy one, as most of the cervical margins we see are in dire need of "protection" of some sort). There are reasons for the preference shown soft foil in the two places mentioned, namely, its easy working properties, the rapidity with which the starting of the filling can be accomplished, and the general wholesale manner in which the cervical margin can be "protected" and the deepest and most inaccessible portion of the cavity filled, placed out of sight, and disposed of.

Of course there are cavities which have no cervical margins and practically no inaccessible portions. I refer to the box-like cavities having four walls, and these are the only cavities where fillings made entirely of soft foil are now, I believe, ever advised; but even in these simplest of all cavities I still think that cohesive gold is far the better material to use.

We are all familiar with the soft gold fillings in crowns and buccal surfaces of short yellow molars, that have been in, so our patients tell us (and I have not the slightest doubt that they speak truly), "ever since s'teen years before old Dr. So-and so-died, who was a fine dentist, but awfully rough." These fillings have done great service, and all honor to the men who put them in; but how about it when we come to proximal cavities in the bicuspids?

There are few practicing dentists who have not had to remove gold fillings from these cavities; and in so doing, how often do we take out of the grinding surface a hard nugget of cohesive gold, and then from the cervical third of the cavity dig out a mass of soft gold that seems to be somehow disintegrated and like so much yellow sausage. Again, how often have we seen soft gold fillings that are battered out of shape, rough, uneven, pitted, discolored, and a disfigurement to the tooth. The cohesive filling is more dense, is harder and wears longer, is susceptible of and will retain a higher finish than soft gold ever can.

The objections to the use of cohesive gold are chiefly its difficulty of manipulation, length of time required for its insertion, necessity of keeping the tooth perfectly dry during the entire operation, the alleged difficulty of making a good adaptation to the cavity walls, expense and discomfort to the patient. Were it not for these few objections, cohesive gold would be almost the only material used for filling teeth to-day.

Unquestionably it often is a difficult matter to start a filling with cohesive gold, that is to get the first few pieces firmly anchored in place. Especially is this true if we try to make the start with foil and the cavity happens to be one where we can see nothing except in the mirror. Happily we have cylinders, mat and sponge gold to help us out of this difficuty. These are all true forms of cohesive gold, but owing to the peculiar properties imparted by the processes of their manufacture, can be gently thrust into the cavity where we can coax them into undercuts and retaining grooves and condense them at our leisure. I use very little mat gold, and never depend on it for any anchorage strength, simply a very tiny piece at the start for the purpose of saving time. Any filling may be started with cohesive foil, if our skill and patience are adequate.—*Dental Brief.*

